# Identification of a novel reactive oxygen species (ROS)-related genes model combined with RT-qPCR experiments for prognosis and immunotherapy in gastric cancer

**DOI:** 10.3389/fgene.2023.1074900

**Published:** 2023-04-14

**Authors:** Kenan Cen, Zhixuan Wu, Yifeng Mai, Ying Dai, Kai Hong, Yangyang Guo

**Affiliations:** ^1^ The Affiliated Hospital of Medical School of Ningbo University, Ningbo, Zhejiang, China; ^2^ First Affiliated Hospital of Wenzhou Medical University, Wenzhou, Zhejiang, China

**Keywords:** reactive oxygen species, tumor microenvironment, prognosis, gastric cancer, immunotherapy

## Abstract

Reactive oxygen species play a crucial role in the prognosis and tumor microenvironment (TME) of malignant tumors. An ROS-related signature was constructed in gastric cancer (GC) samples from TCGA database. ROS-related genes were obtained from the Molecular Signatures Database. Consensus clustering was used to establish distinct ROS-related subtypes related to different survival and immune cell infiltration patterns. Sequentially, prognostic genes were identified in the ROS-related subtypes, which were used to identify a stable ROS-related signature that predicted the prognosis of GC. Correlation analysis revealed the significance of immune cell iniltration, immunotherapy, and drug sensitivity in gastric cancers with different risks. The putative molecular mechanisms of the different gastric cancer risks were revealed by functional enrichment analysis. A robust nomogram was established to predict the outcome of each gastric cancer. Finally, we verified the expression of the genes involved in the model using RT-qPCR. In conclusion, the ROS-related signature in this study is a novel and stable biomarker associated with TME and immunotherapy responses.

## 1 Introduction

Gastric cancer (GC) is associated with high morbidity and mortality rates (Smyth et al., 2020). Surgical resection of advanced GC is the only truly effective treatment (Berretta et al., 2016; van Boxel et al., 2019). However, GC patients have a higher rate of recurrence and metastasis after surgery (Catalano et al., 2005). Therefore, investigating the mechanisms that govern the occurrence and metastasis of gastric cancer, as well as identifying diagnostic markers and therapeutic targets, has become a hotspot.

Reactive oxygen species (ROS) are defined as oxygen-containing reactive species, such as superoxide anions (O2-) and hydroxyl radicals (OH-) (Collin, 2019). Normal levels of ROS participate in multiple signal transduction pathways and control cell proliferation, differentiation, and growth (Mittler, 2017). It appears that a redox imbalance in which ROS production exceeds the cellular capacity to scavenge ROS leads to oxidative stress (Pizzino et al., 2017). This phenomenon has been implicated in the development of GC. In previous studies, ROS have been found to influence GC progression through autophagy (Liu J. Z. et al., 2020; Cao et al., 2020). However, ROS-related genes have not yet been comprehensively identified as playing a significant role in GC.

NOS3 (endothelial NOS, eNOS) is an enzyme belonging to the family of nitric oxide synthases that mainly generates nitric oxide (NO) (Tenopoulou and Doulias, 2020). NO is involved in ROS-mediated malignancy and normal tissue damage (Nissanka and Moraes, 2018). Previous research has shown that NOS3 inhibits apoptosis and promotes tumor proliferation, invasiveness, and immunosuppression (Sun et al., 2020). Therefore, we proposed that the ROS-related genes can be used to predict tumor survival and immunotherapy response.

In our study, based on a systematic investigation of the expression level and clinical characteristics of ROS-related genes, we constructed a prognostic model for these genes in GC patients. In addition, the correlation between these genes and immune cell infiltration was also demonstrated. The prognostic value of these genes can predict tumor immune microenvironment in GC. Furthermore, clinical and immunological characterization of ROS-related genes will be beneficial for the optimization of immunotherapy in GC.

## 2 Methods and materials

### 2.1 Extraction and processing of gastric cancer data

The whole steps performed in the analysis were displayed in the Supplementary Figure S1 The transcription profile and clinical characteristics of gastric cancer were extracted from The Cancer Genome Atlas (TCGA) (https://portal.gdc.cancer.gov/) and the National Center for Biotechnology Information Gene Expression Omnibus (GEO) databases (https://www.ncbi.nlm.nih.gov/geo/). The GEO datasets that contain more than 200 human samples and have complete expression data and clinical information were selected as independent test cohorts. Finally, the GSE84433 as well as the GSE84437 which is a SuperSeries that composed of the GSE84426 and GSE84433 Subseries were chosen. The TCGA-STAD project containing 375 tumor and 32 normal tissues, GSE84437 containing 433 human samples, and GSE84433 containing 357 human tissues were selected for further analysis (Yoon et al., 2020). The transcription profile was converted into fragments per kilobase million (FPKM), and the batch effect of the data in GSE84437 was eliminated. The clinical information of TCGA and GEO datasets were presented in Supplementary File S2. The transcriptomic data of TCGA-STAD was downloaded using the “TCGAbiolinks” R package. The expression difference between tumor and normal tissues was identified using the “limma” algorithm and presented with a heatmap and a volcano plot (Ritchie et al., 2015). The log2 fold-change (log2FC) > 1 and false discovery rate (FDR) < 0.05 were set as the cut-off to screen the differently expressed genes (DEGs). ROS-related genes (Supplementary File S1) were extracted from the Molecular Signatures Database (MSigDB) database (http://www.gsea-msigdb.org/gsea/index.jsp).

### 2.2 Inner correlation between ROS-related genes

A protein-protein interaction (PPI) network of ROS-related genes was constructed using the STRING database (http://string-db.org/), revealing their putative connections. Then, the correlation between these genes and their mRNA expression was further explored by R packages “igraph” and “reshape2 (Csardi and Nepusz, 2006)”. Red lines show positive correlations, whereas blue lines indicate negative correlations.

### 2.3 Identification and verification of ROS-related clusters

First, the consensus clustering was conducted with the expression of prognostic ROS-related genes from the “limma” as well as “ConsensusClusterPlus” R packages (Wilkerson and Hayes, 2010; Ritchie et al., 2015). Through consistent cluster analysis, we obtained a cluster Max K value = 9 and cluster Num = 2. Then, K-M analysis of ROS-related clusters was conducted using the “survival” and “survminer” R packages (Rich et al., 2010). Additionally, a heatmap was applied to show the relationship between clusters and age, sex, tumor grade, clinical and T, N, and M stages, as well as the expression profiles of these genes in different clusters, demonstrating the success of this clustering. Sequentially, the immune cell infiltrations between different clusters were evaluated utilizing the “MCPcounter” R package, including B linage, CD8^+^ T cell, cytotoxic lymphocyte, fibroblasts, T cell, NK cell, monocytic linage, myeloid dendritic cell, and neutrophils (Becht et al., 2016).

### 2.4 Establishment of ROS-related signature

TTCGA-STAD was used as the training set. The GSE84437 and GSE84433 were used as the validation sets. DEGs were identified in ROS-related clusters. Then, least absolute shrinkage and selection operator (LASSO) Cox regression was applied with the DEGs in the TCGA-STAD project to obtain a ROS-related signature (Tibshirani, 1997). The risk score was derived using the formula = $\sum_{i=1}^{n}Coef\left(i\right)*Expr\left(i\right)$ . Univariable Cox regression analyses of genes were conducted to assess the prognostic value of each single gene, and the mutation profiles of these genes were presented using a waterfall plot. The “maftools” R package was used to analyze and visualize the mutation including the missense mutation, non-sense mutation, frame-shift mutation, and multi-hit mutation. Patients were grouped into low- or high-risk subgroups according to the median score of the entire GC. Scatter plots were visualized to establish the correlation between survival status and risk score. Principal component analysis (PCA) and t-distributed stochastic neighbor embedding (t-SNE) analysis were applied to assess the capability of the risk score to distinguish low- and high-risk patients (Ringnér, 2008; Cieslak et al., 2020). The receiver operating characteristic (ROC) curves were conducted to evaluate the predictive ability of the signature by “SurvivalROC” R package (Mandrekar, 2010). K-M survival analyses were conducted between different risk GC groups to verify differences in survival. Besides, three other signatures were used to conduct a comparation analysis with the ROS-related signature, comparing the C-index (Liu et al., 2022; Mak et al., 2022; Zhou et al., 2022).

### 2.5 Development and validation of a ROS-related nomogram

Uni- and multivariable Cox regression analyses were performed on TCGA-STAD and GSE84437 datasets to identify independent prognostic factors. Sequentially, the relationship between the risk score and clinical characteristics was presented in the heatmap. Then we constructed a prognostic nomogram using the “rms” R package (Iasonos et al., 2008). Calibration plots were used to assess the discriminative power of the nomogram (Van Calster et al., 2016). The accuracy of this nomogram was verified using ROC and decision curves (Mandrekar, 2010; Kerr et al., 2016).

### 2.6 Subgroup analyses and immune cell infiltration pattern

The limma algorithm was used to reveal the distinct risk of GC in different subgroups, including age, sex, tumor grade, clinical and T, N, and M stages, as well as immune subtypes (Ritchie et al., 2015). Moreover, Survival differences in the low- and high-risk subgroups were established using the K-M analysis. The ImmuneScore, StromalScore, and EstimateScore were assessed by “estimation of stromal and immune cells in malignant tumor tissues using expression data (ESTIMATE)” algorithm, as well as immune cell infiltrations were evaluated using the “tumor immune estimation resource (TIMER),” “cell-type identification by estimating relative subsets of RNA transcripts (CIBERSORT),” “CIBERSORT-absolute mode (ABS),” “QUANTISEQ,” “microenvironment cell populations-counter (MCPCOUNTER),” “XCELL,” and “estimating the proportion of immune and cancer cells (EPIC)” algorithms (Becht et al., 2016; Aran et al., 2017; Li et al., 2017; Chen et al., 2018; Plattner et al., 2020). In addition, single-sample gene set enrichment analysis (ssGSEA) and immune function analyses have been conducted for different risk patients (Zhu L. et al., 2021).

### 2.7 Prediction of immunotherapy response

The expression of immune-related genes was assessed using the “limma” algorithm. The tumor mutation burden (TMB), microsatellite instability (MSI), and tumor immune dysfunction and exclusion (TIDE) scores were then calculated. TMB score was conducted by R package “maftools” (Mayakonda et al., 2018), MSI score was obtained from previous research (Bonneville et al., 2017), and TIDE score was conducted by online database (http://tide.dfci.harvard.edu/). Finally, the immunophenoscore (IPS) of the different risks was evaluated in different subgroups. IPS refers to four main parts (effector cells, immunosuppressive cells, MHC molecules, and immunomodulators) determining the immunogenicity, and is calculated without bias using machine learning methods. The IPS of STAD patients were downloaded from The Cancer Immunome Atlas (TCIA) (https://tcia.at/home).

### 2.8 Correlation between ROS-related signature and gene mutation and drug sensitivity

To further explore the relevant factors of the ROS-related signature, we established the risk score in distinct wild and mutated GC types, including ARID1A, CSMD3, FAT4, FLG, LRP1B, MUC16, SYNE1, TP53, and TTN. Afterward, the relationship between signature and 5-fuorouracil, gemcitabine, cytarabine, dasatinib, etoposide, GSK690693, masitinib, and tipifarnib were evaluated according to inhibitory concentration (IC50) by the “pRRophetic” R package (Geeleher et al., 2014) based on the Genomics of Drug Sensitivity in Cancer database (GDSC, https://www.cancerrxgene.org/).

### 2.9 Functional enrichment analysis

Gene Ontology (GO) and Kyoto Encyclopedia of Genes and Genomes (KEGG) enrichment analyses were performed to evaluate the functions of DEGs in low- and high-risk GC (Kanehisa and Goto, 2000; The Gene Ontology Consortium, 2019). Gene set enrichment analysis (GSEA) was applied between different risk groups. The threshold of GSEA analysis was log2FC > 1 and *p* < 0.05, and the top five enriched pathways were identified (Subramanian et al., 2005). The “c2. cp.kegg.v7.4. symbols. gmt” file downloaded from the GSEA database (https://www.gseamsigdb.org/gsea/index.jsp). Moreover, gene set variation analysis (GSVA) was performed with reference to the methods of previous research, and the enriched functions of different risk groups were visualized using a heat map (Hänzelmann et al., 2013). The enrichment analyses were performed by the “limma” and “GSVA” R packages (Yu et al., 2012; Ritchie et al., 2015).

### 2.10 Cell culture

HGC-27 and GES-1 cells were obtained from the Cell Bank of Shanghai Institute of Biochemistry and Cell Biology (Shanghai, China). The cell lines were cultured in DMEM or RPMI-1640 supplemented with 10% fetal bovine serum (FBS), 100 μg/mL streptomycin, and 100 U/mL penicillin (Gibco).

### 2.11 Clinical sample

All samples were obtained with the approval of the Ethics Committee of Ningbo First Hospital. A total of 4 GC patients who signed the informed consents were recruited from the Ningbo First Hospital. The tumor samples and normal tissues were collected from the surgical specimen of the patients.

### 2.12 RT-qPCR

Total RNA was extracted using TRIzol reagent (Invitrogen) and reverse-transcribed into cDNA templates. β-Actin was used as an endogenous reference. The primer sequences used for amplification are listed in Supplementary File S3.

### 2.13 Statistical analysis

All statistical analyses were performed using the R software (version 4.1.3) (Supplementary File S4). Group comparisons were performed using the Wilcoxon test or Student’s t-test. Correlations between two continuous variables were assessed using Spearman’s correlation analysis. The Kruskal-Wallis test was used to compare the three groups. Adjusted *p*-value was calculated using Benjamini-Hochberg FDR. Statistical significance was set at *p* < 0.05.

## 3 Results

### 3.1 Expression and PPI network of ROS-related gene

**FIGURE 1:**
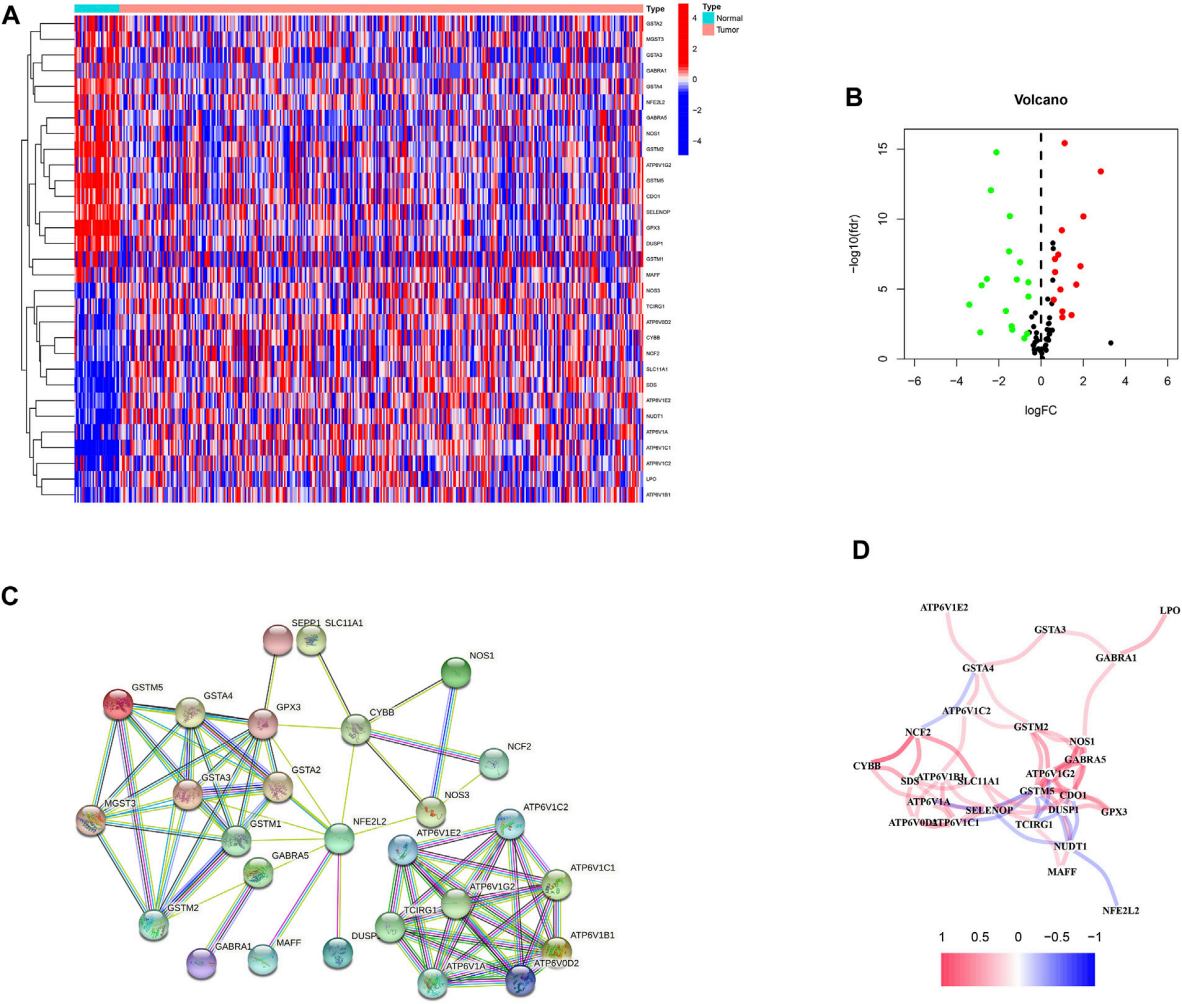
ROS-related genes in GC. **(A)** A heatmap of differentially expressed ROS-related genes in GC. **(B)** A volcano plot of ROS-related genes in GC. **(C)** A protein-protein interaction network. **(D)** A correlation network of ROS-related genes.

First, 87 ROS-related genes were obtained from the GSEA database. Using the “limma” package, 31 differentially expressed ROS-related genes were identified (Figures 1A, B). The PPI network was constructed to identify ROS-related DEG interactions (Figure 1C). Meanwhile, Figure 1D shows the correlation network of DEGs, in which different colors indicate different correlation coefficients.

### 3.2 Identification of ROS-related molecular subtypes

**FIGURE 2:**
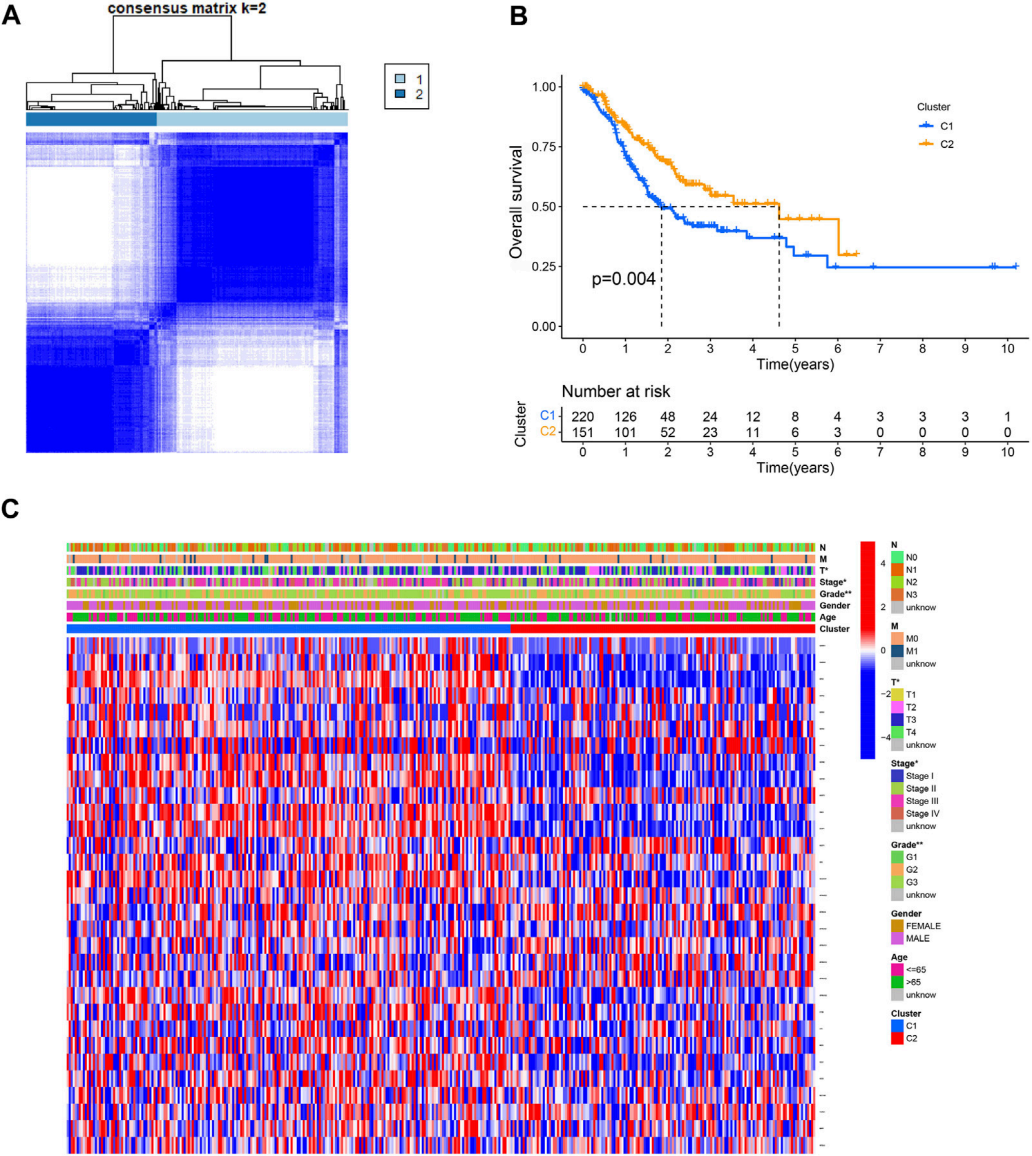
Consensus clustering of ROS-related genes in GC. **(A)** Clustering heatmap for k = 2. **(B)** K-M curve of C1 and C2. **(C)** A heatmap for correlation of clusters with clinical characteristics.

**FIGURE 3:**
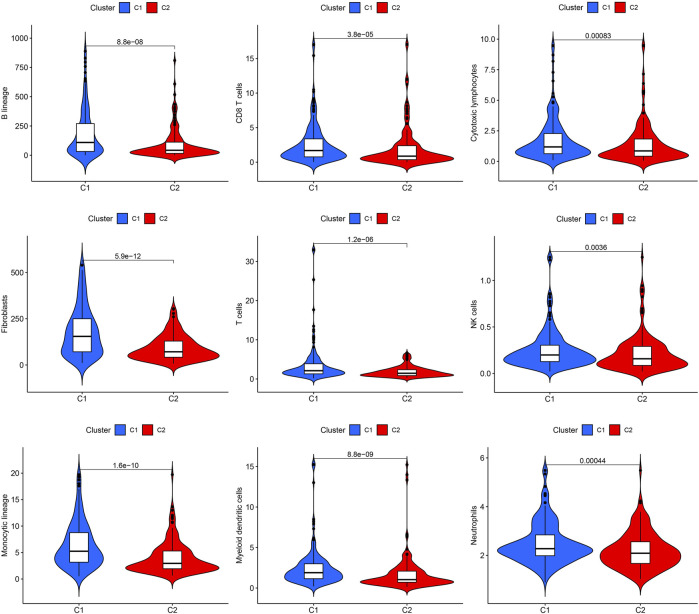
Immune cell infiltrations of cluster 1 and 2.

The effect of ROS-related genes on GC samples was assessed using consensus clustering analysis. When k = 2, the cumulative distribution curve was most horizontal in the middle section and the heatmap of clustering showed a clear edge. Therefore, GC samples in TCGA-STAD were grouped into two clusters based on ROS gene expression (Figure 2A). The overall survival (OS) rates of the C1 and C2 clusters were significantly different, and the prognosis of the C1 cluster was worse than that of the C2 cluster (Figure 2B). Besides, the K-M survival curve of progression free survival (PFS) also demonstrated the poorer outcome of C1 cluster (Supplementary Figure S2). Figure 2C shows the gene expression profiles between the C1 and C2 clusters. We also analyzed differences in immune cell infiltration between the two clusters and found more immune cell infiltration in the C1 cluster, including B lineage, CD8 T cells, cytotoxic lymphocytes, T cells, NK cells, neutrophils, fibroblasts, monocytic lineage, and myeloid dendritic cells, than in the C2 cluster (Figure 3).

### 3.3 Establishment and validation of the signature

**FIGURE 4:**
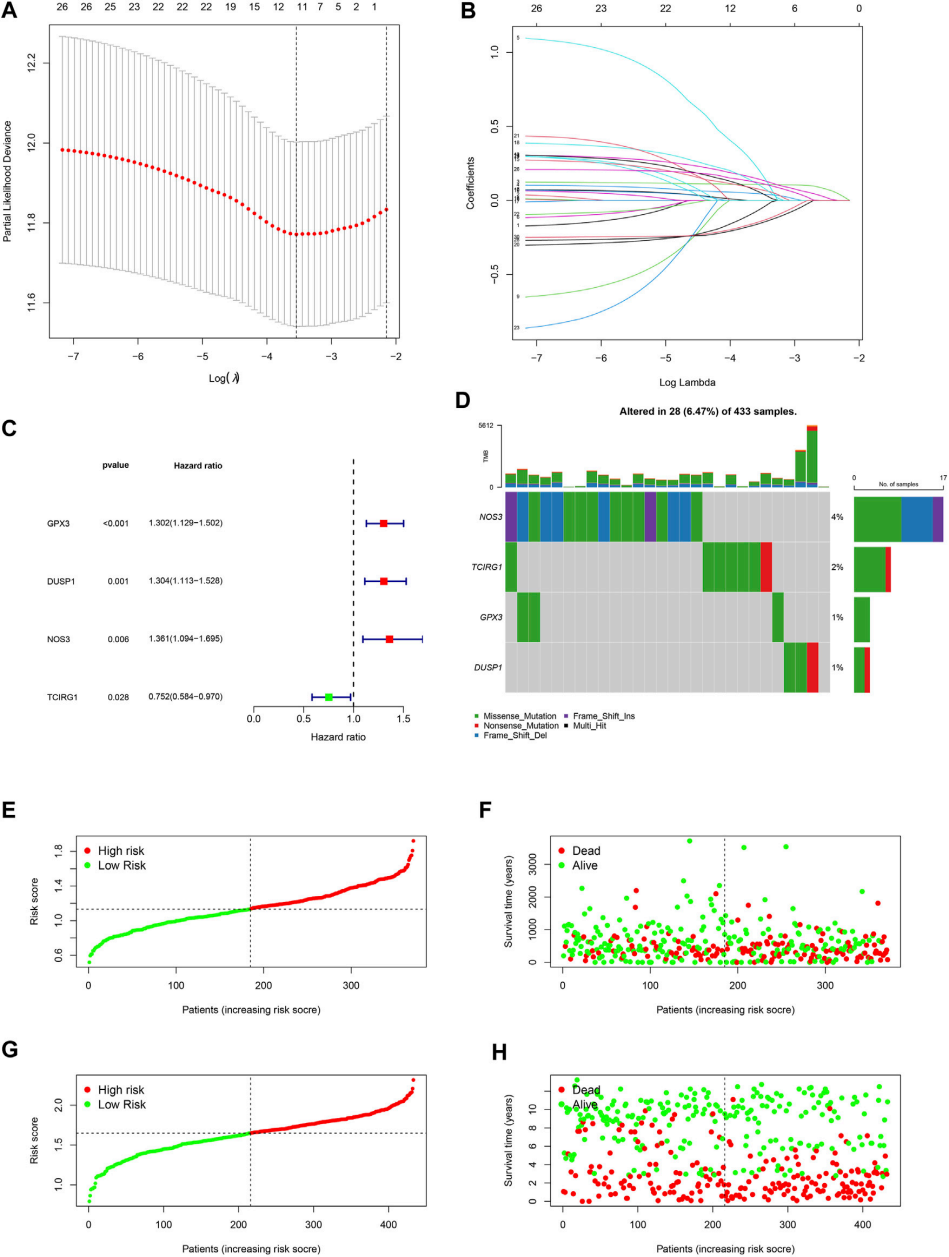
Development of a ROS-related signature in GC. **(A)** A univariable Cox regression analysis of genes constructing the signature. **(B)** A waterfall of mutation profile of genes constructing the ROS-related signature. **(C)** LASSO regression of prognostic genes of cluster 1 and 2. **(D)** Cross-validation for tuning parameter selection. **(E)** Risk score of GC in TCGA-STAD dataset. **(F)** Correlation of survival status with risk score in TCGA-STAD dataset. **(G)** Risk score of GC in GSE84437 dataset. **(H)** Correlation of survival status in GSE84437 dataset.

**FIGURE 5:**
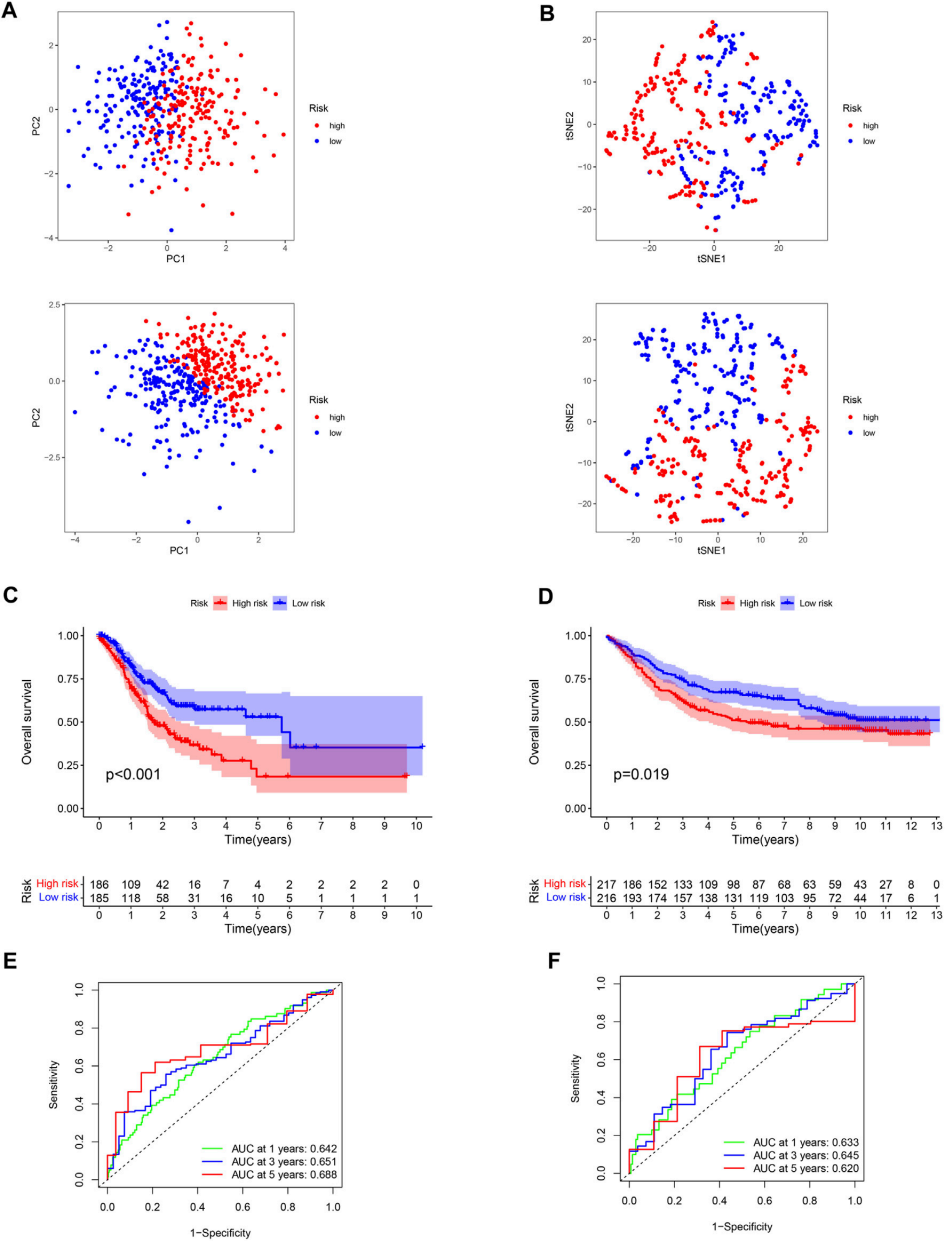
Verification of the stability of ROS-related signature. **(A)** PCA analysis of low- and high-risk GC for TCGA-STAD and GSE84437 datasets. **(B)** t-SNE analysis for TCGA-STAD and GSE84437 datasets. **(C)** K-M curve for different risk GC in TCGA-STAD dataset. **(D)** K-M curve for patients in GSE84437 dataset. **(E)** ROC analysis of the ROS-related signature across TCGA-STAD project. **(F)** ROC analysis of ROS-related signature across GSE84437 dataset.

A 4-gene prognosis model including GPX3, DUSP1, NOS3, and TCIRG1 was constructed using LASSO Cox regression (Figures 4A, B). All of the signature genes were identified prognostic genes by univariable Cox analysis, including GPX3, DUSP1, NOS3, and TCIRG1 (Figure 4C). We also analyzed mutations in these genes in GC (Figure 4D). GC patients were divided into high- and low-risk groups according to the median risk score in the training cohort (Figures 4E, F) and test cohort (Figures 4G, H). Patients in the high-risk group had a higher risk of death and poorer prognosis than those in the low-risk group. As shown in Figures 5A, B, PCA demonstrated clear distinctions between the two risk groups.

**FIGURE 6:**
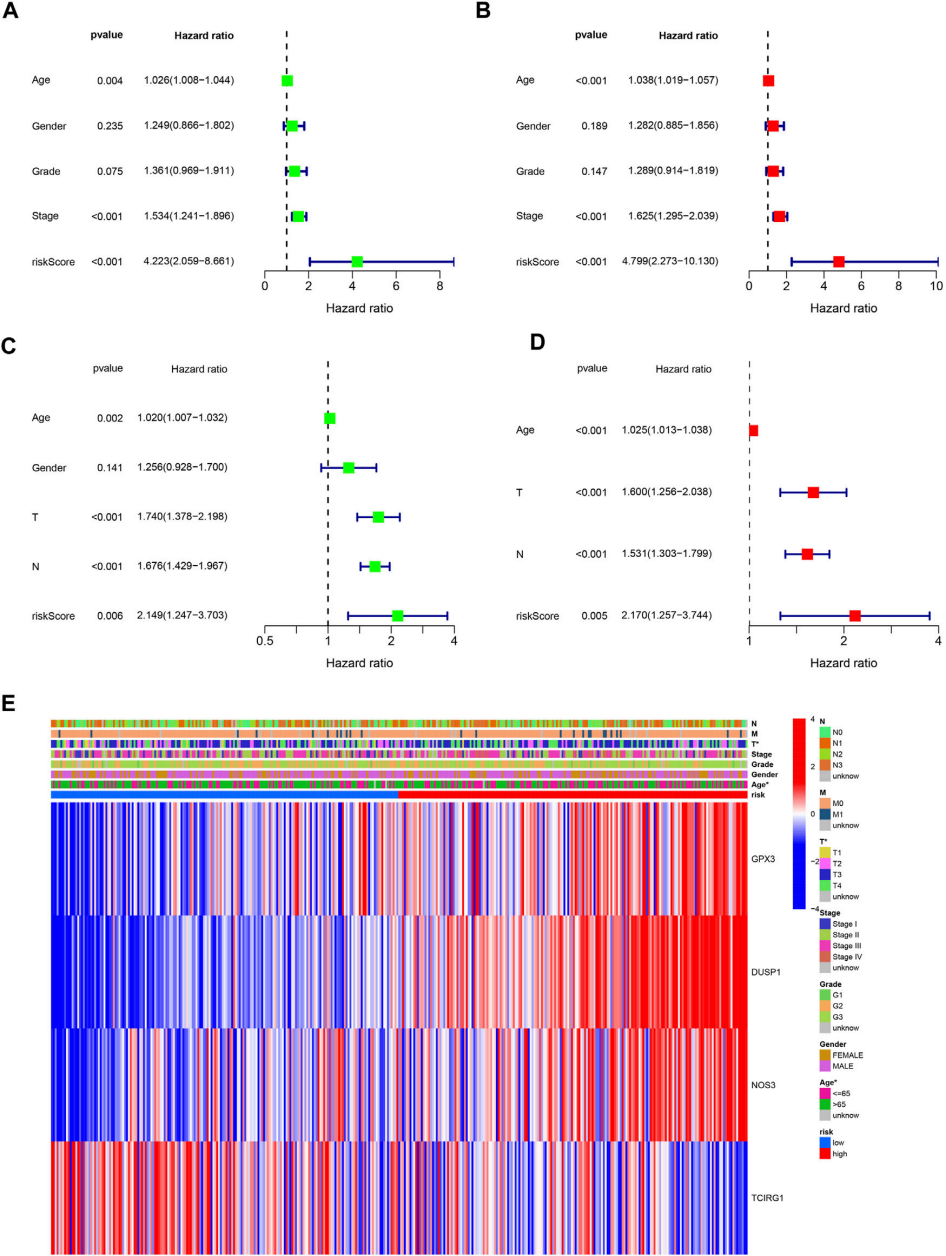
Independently predictive ability of the signature. **(A, B)** Univariable and Multivariable analysis across TCGA dataset. **(C)** Univariable analysis across GSE84437 dataset. **(D)** Multivariable analysis of the signature across GSE84437 dataset. **(E)** A heatmap for the correlation of the signature with clinical characteristics.

Low-risk patients were linked to better prognoses based on survival curves (Figure 5C) in the TCGA cohort. Similarly, in the test cohort, patients with lower-risk scores showed better survival (Figure 5D and Supplementary Figure S3). Additionally, the area under the curve (AUC) value indicated that our model had a good predictive power (Figures 5E, F). According to Cox regression analyses, the risk score was regarded as an independent prognostic factor for the training and testing cohorts (Figures 6A–D). The landscape of the four signature genes in the training group is shown in the heatmap (Figure 6E). The comparation analysis indicated that the stability of our signature is better than signatures of Mak et al., Zhou et al., and Liu et al. (C-index, 0.614 to 0.568, 0.597, and 0.607) (Supplementary Figure S4). To explore the genomic alterations in low- and high-risk GC, gene mutation analysis was conducted. Result showed that the TMB of low-risk GC is significantly higher than high-risk GC. Besides, some vital cancer-related genes such as TTN, TP53, MUC16, and ARID1A more frequently mutated in low-risk GC (Supplementary Figure S5).

**FIGURE 7:**
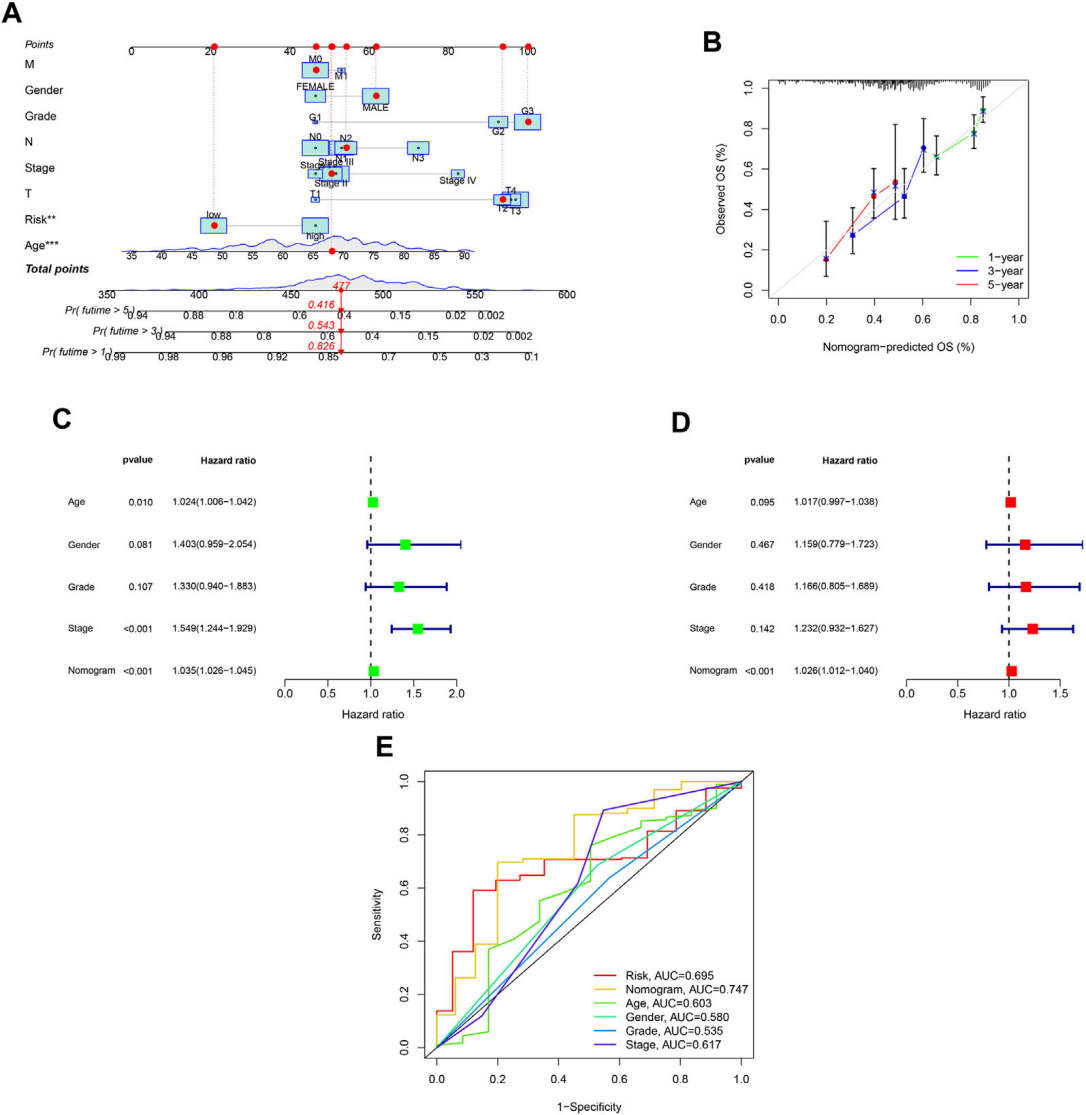
Establishment of a nomogram with ROS-related signature and clinical characteristics. **(A)** A nomogram contains ROS-related signature, age, gender, tumor grade, clinical and T, N and M stage. **(B)** A calibration plot for assessing the predictive power of nomograms at 1-, 3-, and 5-year. **(C)** Univariable analysis of the nomogram across TCGA dataset. **(D)** Multivariable analysis across TCGA dataset. **(E)** DCA analysis of the nomogram model.

A nomogram was developed to predict survival rates in GC (Figure 7A). The predicted results were in good agreement with the actual results according to the calibration plots (Figure 7B). Moreover, the nomogram was regarded as an independent prognostic factor (Figures 7C,D). The AUC of the nomogram was 0.747 (Figure 7E).

### 3.4 Correlation of prognostic model with clinical features

**FIGURE 8:**
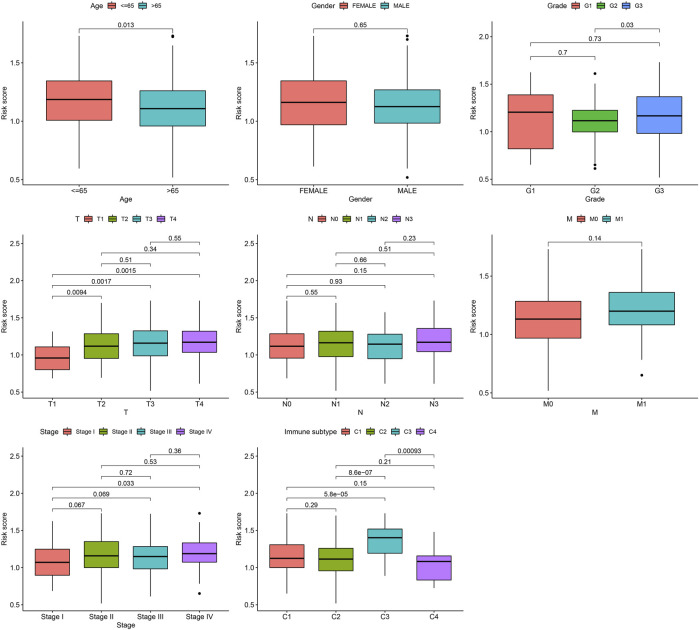
Risk score variation of different risk GC in different subgroups.

**FIGURE 9:**
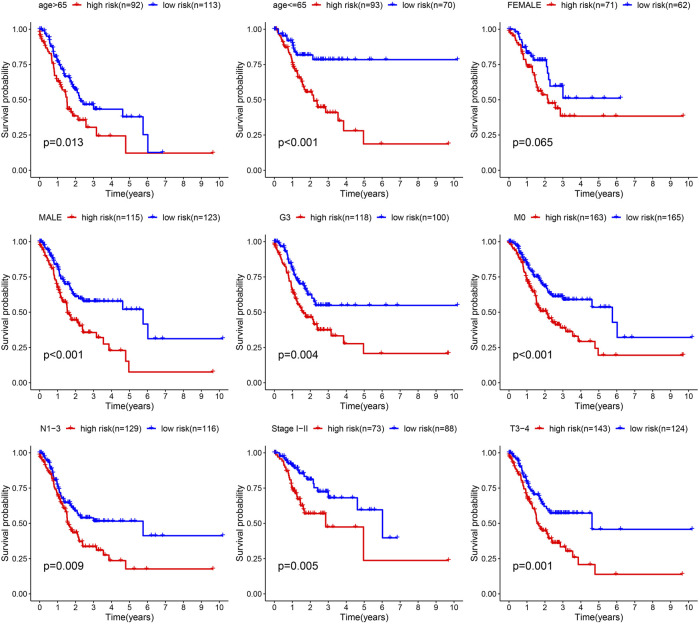
Survival differences of GC in different subgroups.

Next, the correlation between the risk scores and clinical features was studied. Different subgroups, including age, grade, T and TNM stage, as well as immune subtype, had significantly distinct risk scores (Figure 8). In addition, subgroup analysis showed differences in survival between different risk groups in different GC subgroups, including age >65 years, female, male, M0, age ≤65 years, Grade III, stage III-IV, N1-3 and T3+4, further verifying the reliability of this model (Figure 9).

### 3.5 Correlation of the model with immune activity

**FIGURE 10:**
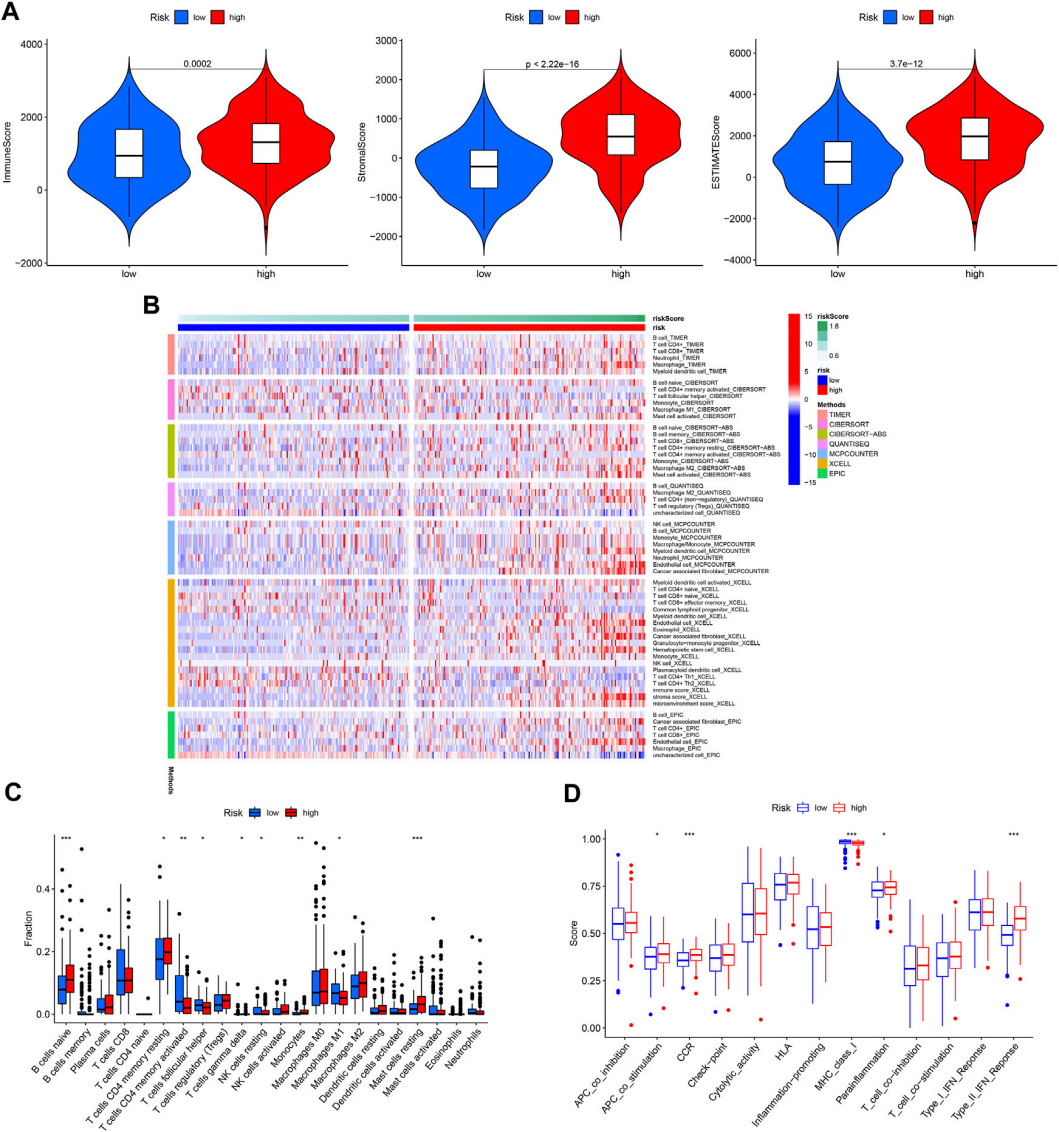
Immune cell infiltration pattern in different risk GC. **(A)** Tumor purity analysis. **(B)** Immune cell infiltration of different risk GC. **(C)** ssGSEA analysis. **(D)** Immune function analysis of different risk GC.

The immune microenvironment plays a critical role in the development of GC. In our study, we found that patients in the high-risk group had higher immune, stromal, and ESTIMATE scores (Figure 10A). Next, the differences in immune cell infiltration between the different risk groups were assessed (Figure 10B). Infiltration of Monocytes, Macrophages M2, Mast cells resting, naïve B cells, and eosinophils was more abundant in the high-risk group, while infiltration of CD4 memory activated T cells, resting NK cells resting and Macrophages M1 was more abundant in the low-risk group (Figure 10C). In addition, APC co-stimulation, CCR, and Type II IFN responses were usually more significant in the high-risk group (Figure 10D).

**FIGURE 11:**
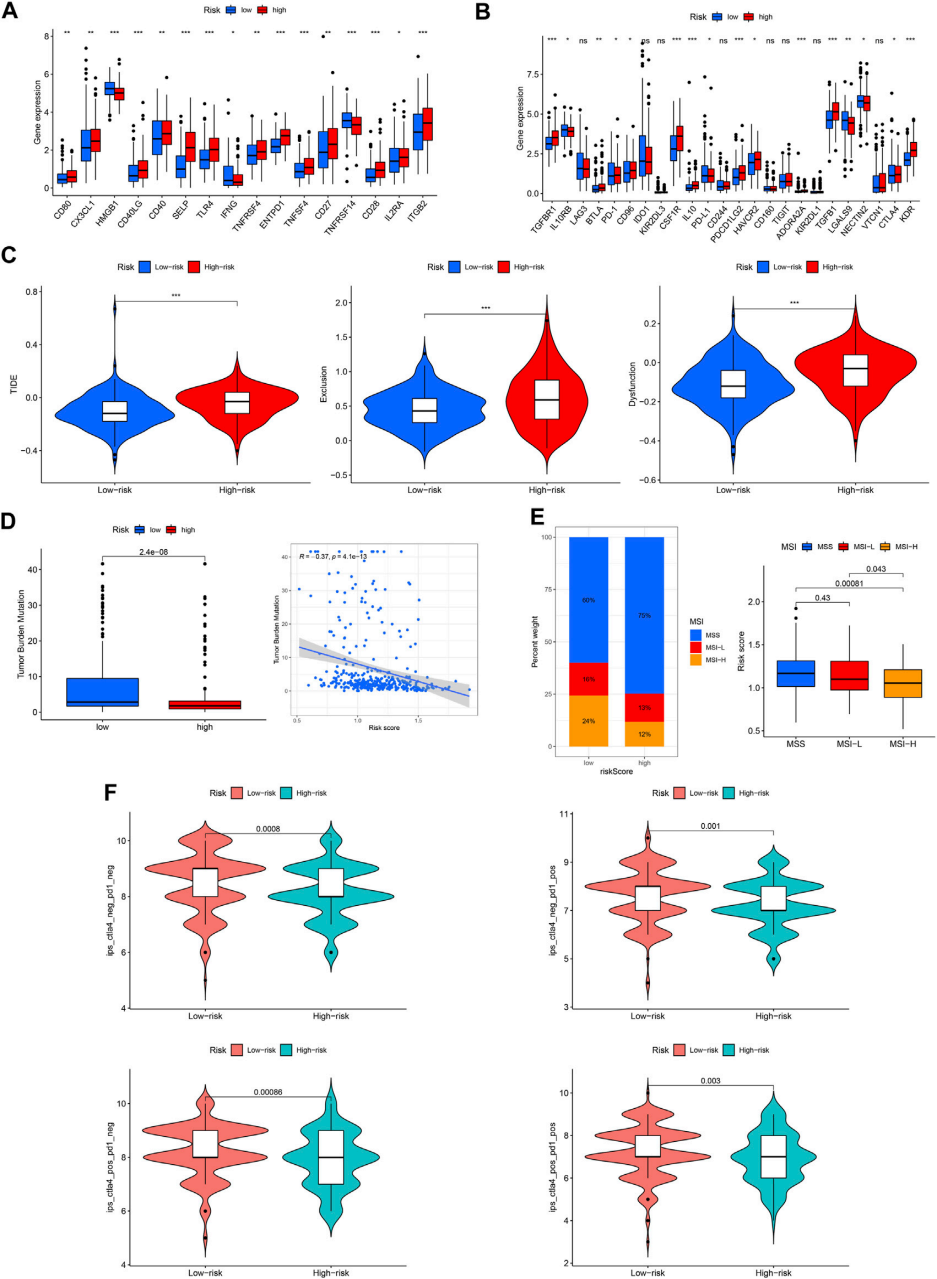
Capacity of immunotherapy response of the ROS-related signature. **(A, B)** Expression difference of immune checkpoint in different risk GC. **(C)** TIDE score. **(D)** TMB score. **(E)** MSI of low- and high-risk GC. **(F)** IPS of low- and high-risk GC in different subgroups.

Immune checkpoint molecules are also involved in the occurrence and development of GC. We found that the expression of most checkpoint genes was higher in the high-risk group (Figures 11A, B). TIDE, TMB, and MSI were used to predict the response to tumor immunotherapy. As shown in Figure 11C, the TIDE, exclusion, and dysfunction scores were all higher in the high-risk group than in the low-risk group. Moreover, the TMB and MSI scores were higher in the low-risk group (Figures 11D, E). Furthermore, we found that patients in the low-risk group responded better to immunotherapy (Figure 11F).

### 3.6 Correlation of prognostic model with genetic mutations and predict chemotherapy drug sensitive

**FIGURE 12:**
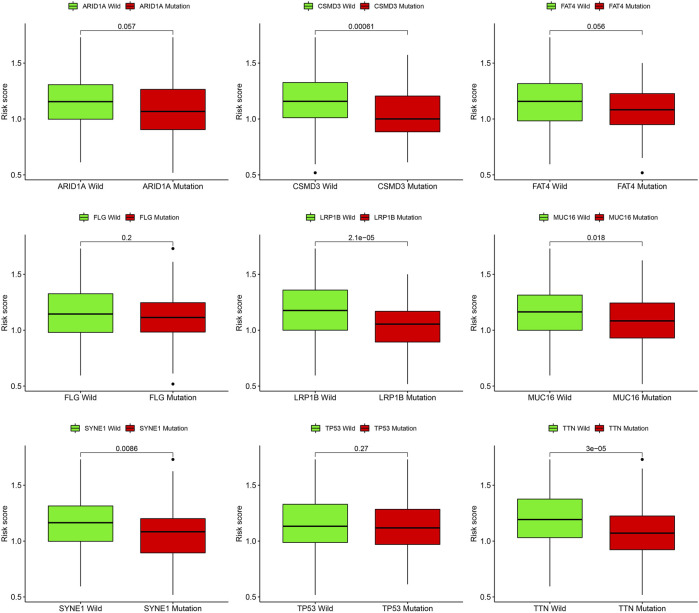
Risk score difference of distinct gene mutation types.

**FIGURE13:**
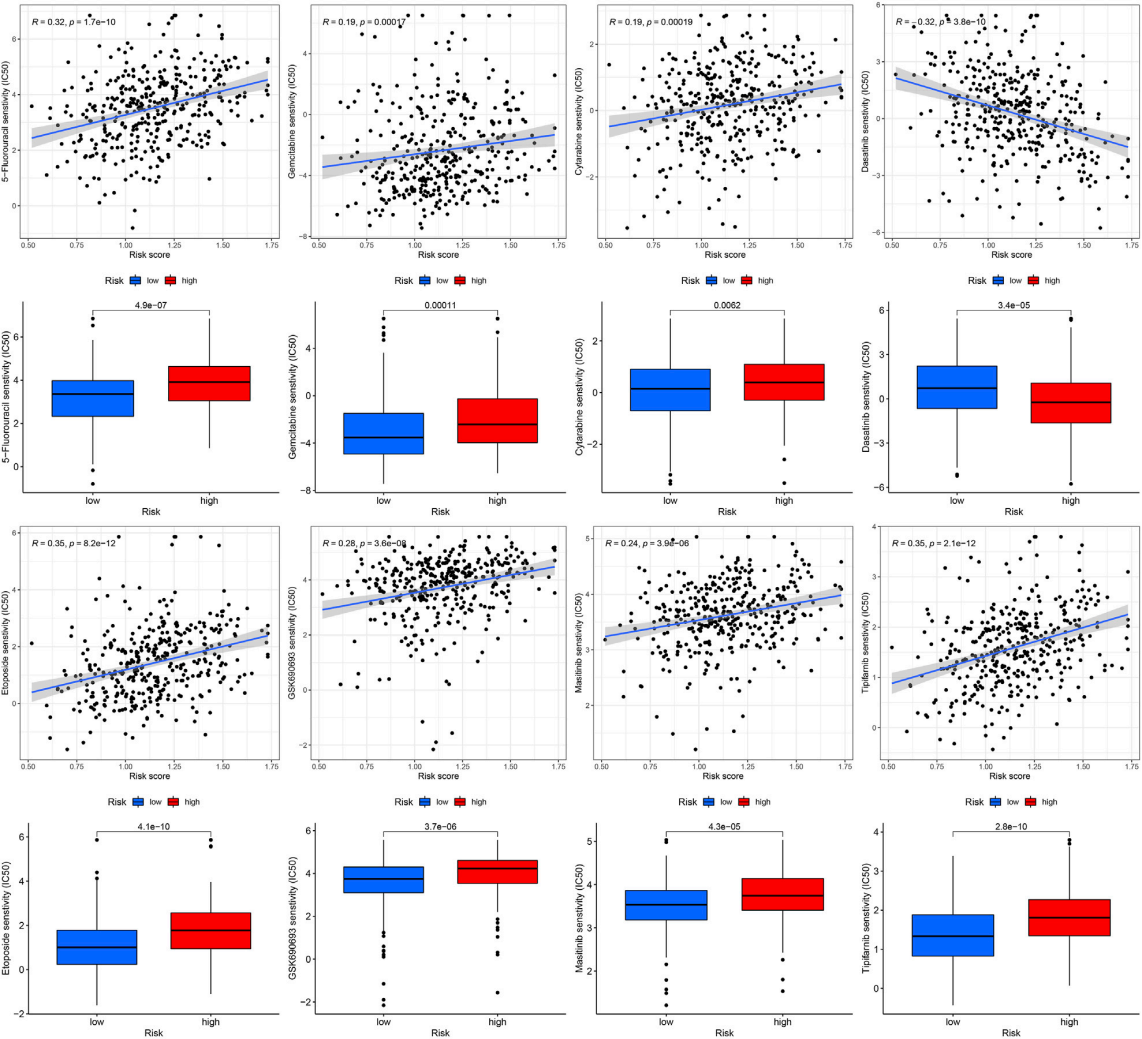
Drug sensitivity of diverse agent in different risk GC.

Gene mutations are crucial factors in tumor development. In the current study, the correlation between prognostic models and genetic mutations was analyzed. We found that the risk score was lower in the mutation group than in the wild-type group (Figure 12). Next, we predicted the chemoresponses of the subgroups to common chemotherapeutics (Figure 13). The results revealed that high-risk patients were more sensitive to 5-Fluorouracil, Gemcitabine, cytarabine, dasatinib, etoposide, GSK690693, masitinib, and tipipifarnib, suggesting that these patients could benefit from these chemotherapeutic agents.

### 3.7 Functional enrichment analysis

**FIGURE 14:**
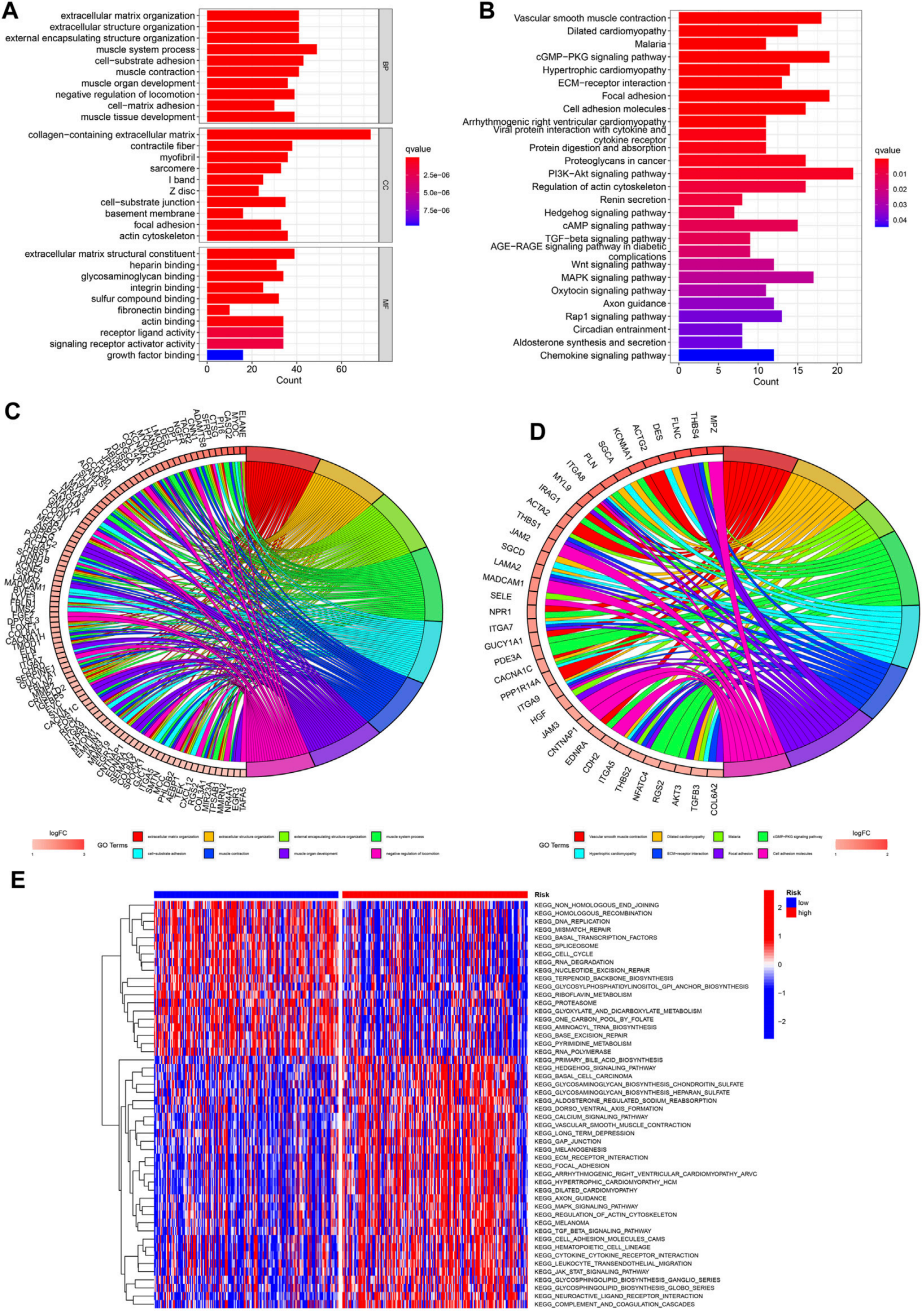
Functional enrichment analysis. **(A. C)** GO analysis of DEGs between low- and high-risk GC. **(B, D)** KEGG analysis. **(E)** GSVA analysis of low- and high-risk GC.

To further explore the functional enrichment of the signature, we performed an enrichment analysis of DEGs between the low- and high-risk groups. GO enrichment indicated that DEGs were enriched in “muscle system process,” “collagen−containing extracellular matrix,” and “extracellular matrix structural constituent” (Figures 14A, C). KEGG enrichment analysis showed significant enrichment of the “PI3K-AKT” and “cGMP-PKG” signaling pathways (Figures 14B, D). Additionally, from the heatmap of GSVA, significant difference of enriched functions between different risk groups were presented (Figure 14E).

### 3.8 RT-qPCR in GC

**FIGURE 15:**
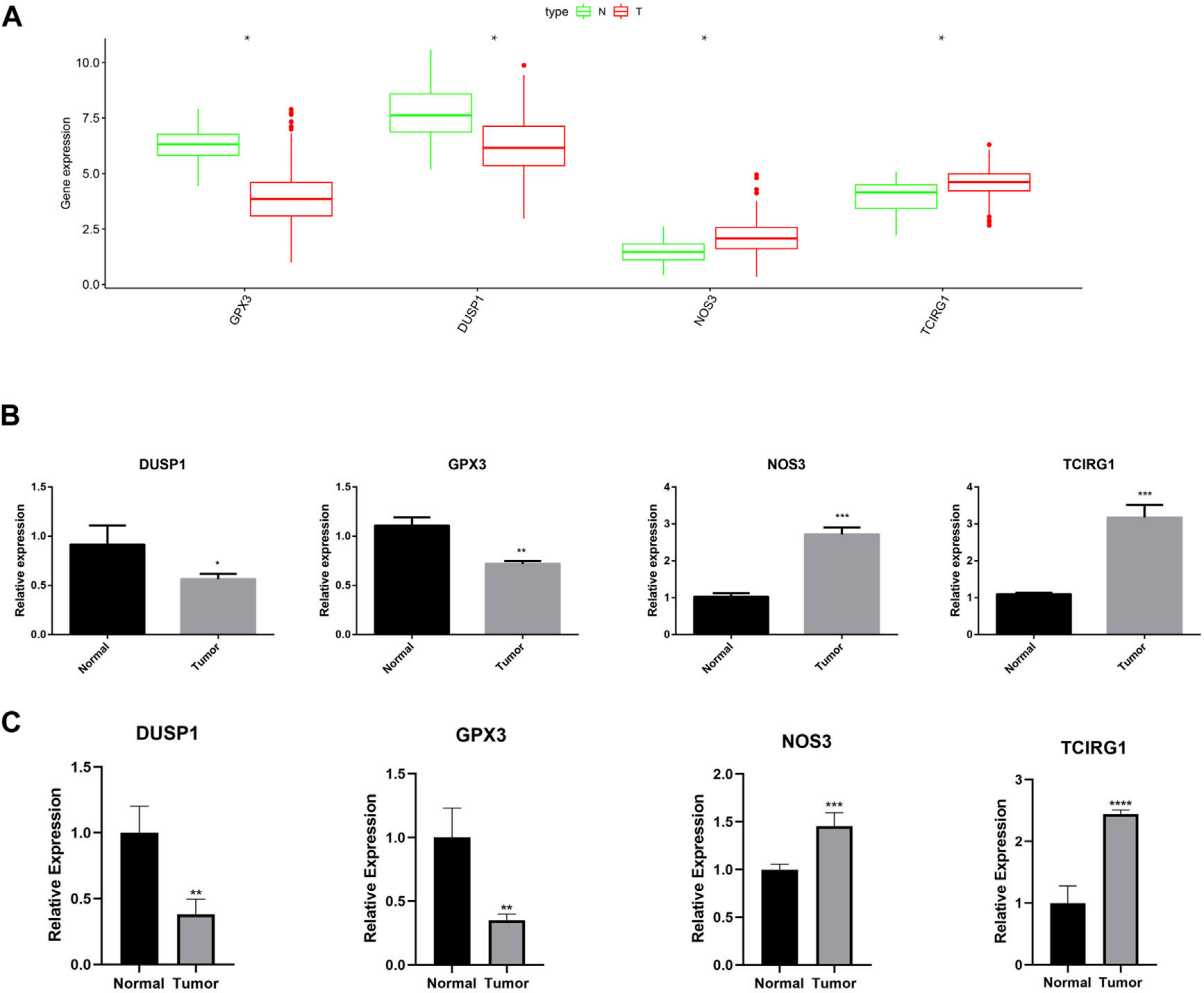
Expression of GPX3, DUSP1, NOS3, and TCIRG1. **(A)** Expression of DUSP1, GPX3, NOS3, and TCIRG1 in GEPIA2 online set. **(B)** RT-qPCR detected the expression of DUSP1, GPX3, NOS3, and TCIRG1 in gastric cancer cell and normal cell. **(C)** RT-qPCR detected the expression of DUSP1, GPX3, NOS3, and TCIRG1 in gastric cancer samples and normal tissues.

As shown in Figure 15A, the expression of GPX3, DUSP1, NOS3, and TCIRG1 was explored using the GEPIA2 site (http://gepia2.cancer-pku.cn/#index). Low expression of GPX3 and DUSP1 was observed in GC tissues as compared to that in normal tissues, whereas NOS3 and TCIRG1 were highly expressed in GC tissues. RT-qPCR was performed to verify the expression levels of these genes. The results demonstrated that NOS3 and TCIRG1 were highly expressed in tumor cells and samples compared to normal cells and tissues, while GPX3 and DUSP1 were expressed at low levels in tumor cells and samples (Figures 15B, C).

## 4 Discussion

With advances in high-throughput sequencing technologies, an increasing number of disease targets and prognostic biomarkers have been identified. However, reactive oxygen species-related prognostic biomarkers for GC remain limited. More and more researches have shown that elevated ROS levels are closely linked to the occurrence and development of cancer (Wang et al., 2019). A prognostic model based on these genes was constructed to elucidate the roles of ROS-related genes in GC. Next, the correlation of the prognostic model with immune-infiltrating cells was demonstrated. In addition, the model showed excellent predictive performance for the immune microenvironment of patients with GC. Furthermore, the clinical and immunological characteristics of the constructed prognostic model for GC may help optimize tumor immunotherapy.

ROS refers to a class of oxygen-containing compounds converted from molecular oxygen, whose chemical properties are more active than molecular oxygen (Wu and Cederbaum, 2003). Living organisms have a complex oxidative-antioxidant system (Birben et al., 2012). Under normal circumstances, ROS maintained within a stable range can play a positive role in anti-inflammatory and antibacterial activities (Zhu et al., 2021). When this balance fails to be maintained, ROS increase, promoting cell transformation and lead to the occurrence of malignant tumors (Kim et al., 2016). Studies have shown that ROS can disrupt mitochondrial function and promote oxidative stress, leading to gastric carcinogenesis (Lee and Yu, 2016).

Our 4-gene (GPX3, DUSP1, NOS3, TCIRG1) signature based on ROS-related genes was used for GC. Previous studies have reported that these genes play crucial roles in various cancers, including GC. For example, GPX3 inhibits the growth and spread of GC by regulating methylation and ROS (Xie et al., 2022). Cheng et al. found that DUSP1 activates the MAPK pathway in GC, leading to apatinib resistance (Teng et al., 2018). Moreover, Zhang et al. revealed that the expression level of OS3 was increased and significantly associated with poor patient prognosis in GC (Zou et al., 2021). In this study, we found that high-risk patients had a poorer prognosis.

ROS are not only associated with tumorigenesis and development but also with immune checkpoint inhibitors. The study has shown that ROS can induce the expression of PD-L1 by regulating JAK/STAT3 pathway (Liao et al., 2021). In our study, we also found that different risk groups had different immune checkpoint expression, and the survival time of patients with high expression of TGFB1, TGFBR1, CD27, BTLA, CD40, and IL-10 in the high-risk group was shorter than that in the low-risk group. In addition, we found that the constructed prognostic model could predict the response to immunotherapy, with patients in the high-risk group having a poorer response to immunotherapy.

The occurrence of tumors is an extremely complicated biological process, and there are various non-tumor cells in its microenvironment, such as cancer-associated fibroblasts (CAF), tumor-associated macrophages (TAM), immune cells and various lymphatic vessels, tissue fluid, and cytokines, which can maintain tumor growth and increase tumor heterogeneity, adaptability, and metastasis, eventually leading to the development of malignant tumors (Chen et al., 2021; Laha et al., 2021; Raskov et al., 2021; Zhou et al., 2021). Our study found a higher degree of macrophage M2 infiltration in the high-risk group, which may contribute to its poor prognosis. M2 macrophages are involved in tumorigenesis, growth, invasion, and metastasis, and often promote tumor cell growth, angiogenesis, and migration (Zhao et al., 2020). Numerous studies have shown that TAM present in tumor tissues usually exhibit an M2-like phenotype, which is closely associated with cancer treatment and prognosis (Liu et al., 2020; Pan et al., 2020).

GO and KEGG analyses based on DEGs between different risk groups were performed to further elucidate the underlying mechanisms of ROS-related genes. GO analysis revealed that the DEGs were enriched in “collagen−containing extracellular matrix,” “muscle system process,” and “extracellular matrix structural constituent.” KEGG showed significant enrichment of “PI3K-AKT” and “cGMP-PKG” signaling pathway. The PI3K-AKT signaling pathway is involved in the occurrence and development of various cancers, and can promote the proliferation and invasion of cancer cells, inhibit apoptosis, and promote tumor angiogenesis, thereby leading to the progression of malignant tumors (Alzahrani, 2019; Ediriweera et al., 2019; Noorolyai et al., 2019).

In addition, we evaluated the effectiveness of some common chemotherapeutic agents in different risk groups and found that patients in the high-risk group were more sensitive to eight chemotherapeutic agents (5-fluorouracil, gemcitabine, cytarabine, dasatinib, etoposide, GSK690693, masitinib, and tipifarnib), indicating that high-risk patients may benefit from these eight chemotherapy drugs.

In general, we have provided the most comprehensive elucidation of ROS-related genes in GC. We first constructed an ROS-related prognostic model for GC, confirmed the relationship between the model and immune checkpoint genes, as well as immune infiltration, and predicted the response of different risk patients to immunotherapy and chemotherapy. It provides a screening tool for the diagnosis and prognosis of gastric cancer, and a new way to dissect the relationship between gastric cancer and immunity. Our tool contained only four genes and had good stability. Comparing to the clinical prediction tools such as 21-gene score and 70-gene for breast cancer, the Ros-related signature had better economic viability and may use to improve the treatment of clinical GC in the future.

## Data Availability

The raw data supporting the conclusion of this article will be made available by the authors, without undue reservation.

## Supplementary material

SUPPLEMENTARY FILE S1 The ROS-related genes extracted from the MSigDB.

SUPPLEMENTARY FILE S2 The clinical information of TCGA and GEO datasets.

SUPPLEMENTARY FILE S3 The primer sequences used for RT-qPCR.

SUPPLEMENTARY FILE S4 Original R code of the analysis.

SUPPLEMENTARY FIGURE S1 The flowchart of the analysis.

SUPPLEMENTARY FIGURE S2 The PFS analysis between C1 and C2 clusters.

SUPPLEMENTARY FIGURE S3 The K-M survival analysis between low- and high-risk GC.

SUPPLEMENTARY FIGURE S4 The comparation analysis between ROS-related signature and other three models.

SUPPLEMENTARY FIGURE S5 The mutation profile of low- and high-risk GC.
