# Supplementary figures and images for: Identification of a novel reactive oxygen species (ROS)-related genes model combined with RT-qPCR experiments for prognosis and immunotherapy in gastric cancer
Source: Front Genet. 2023 Apr 14;14:1074900. doi: 10.3389/fgene.2023.1074900 (PMC10141461; doi:10.3389/fgene.2023.1074900)

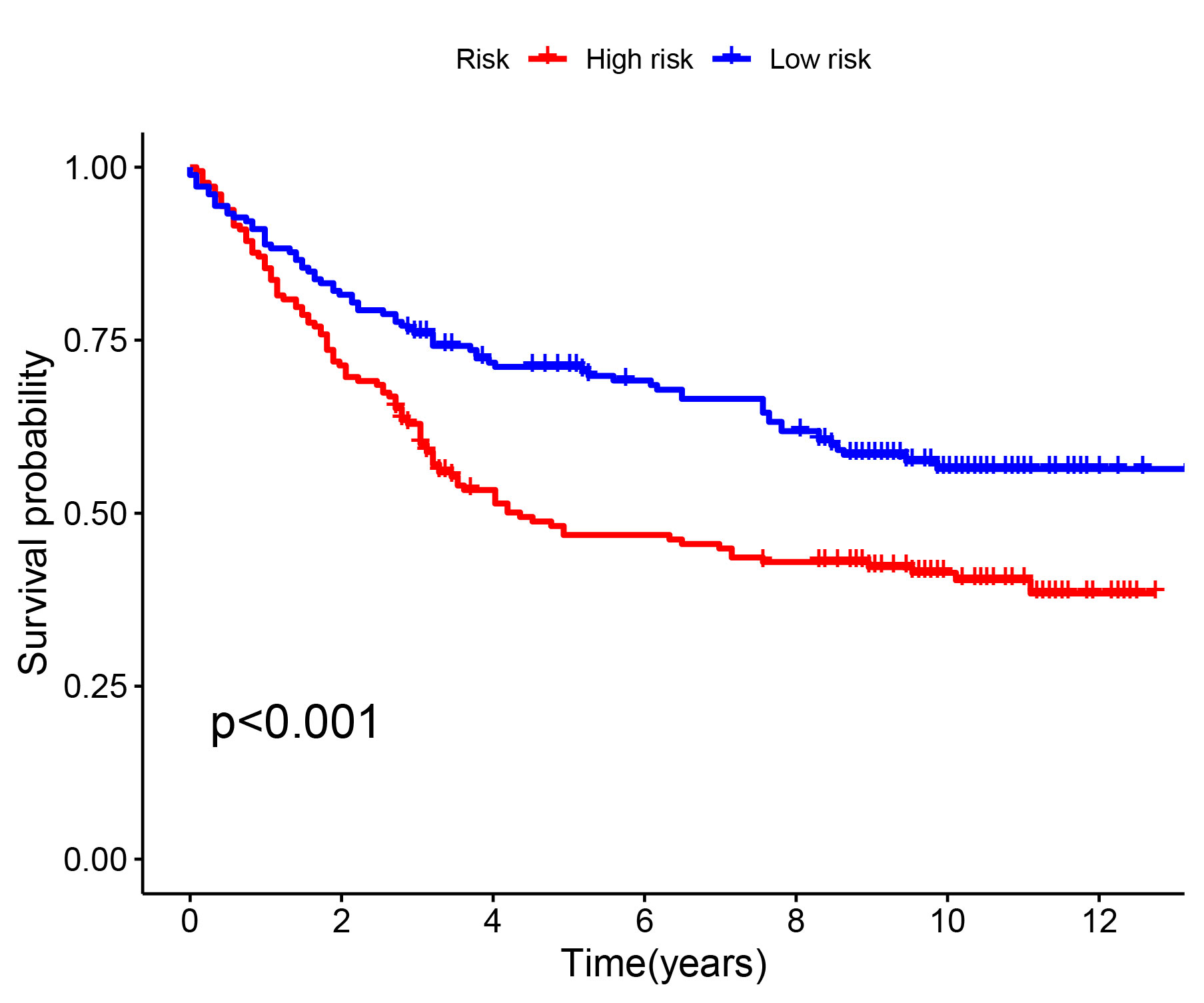

Supplement: Supplementary file 1 [file Image3.JPEG]

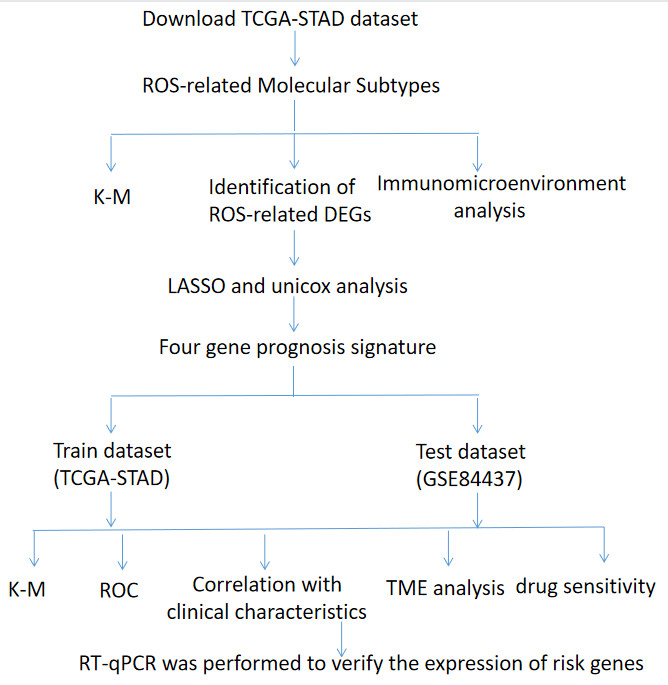

Supplement: Supplementary file 4 [file Image1.JPEG]

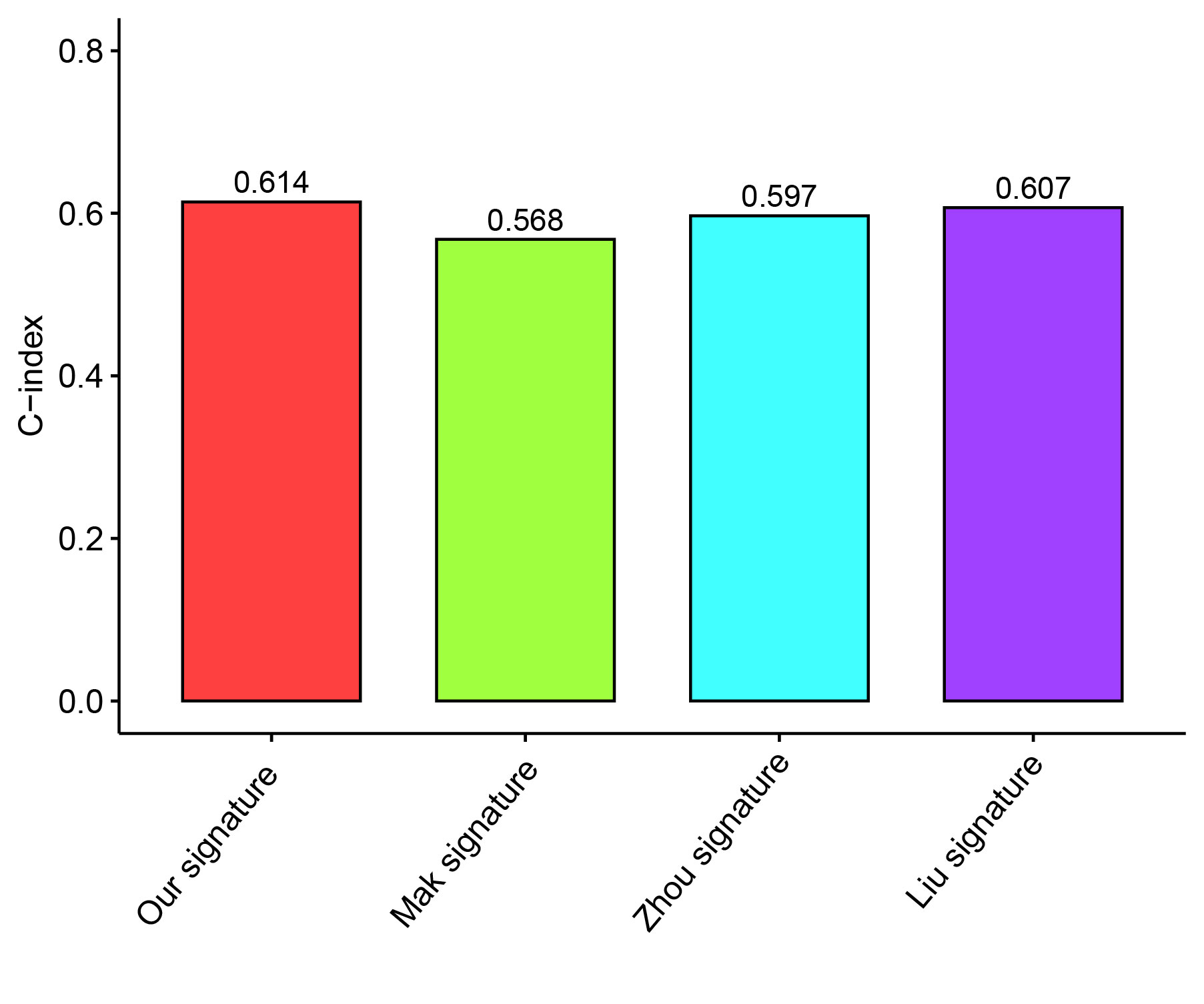

Supplement: Supplementary file 5 [file Image4.JPEG]

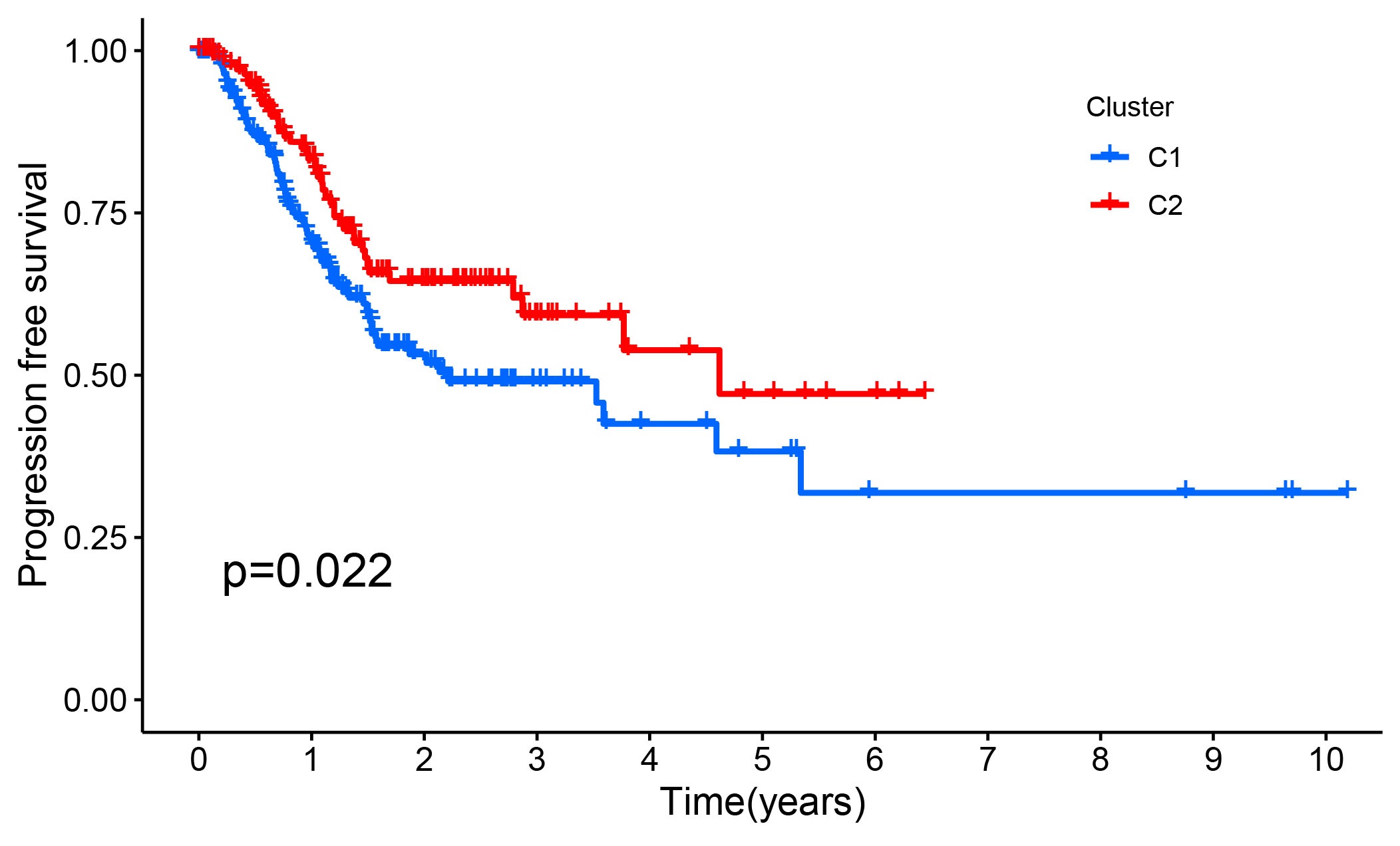

Supplement: Supplementary file 6 [file Image2.JPEG]

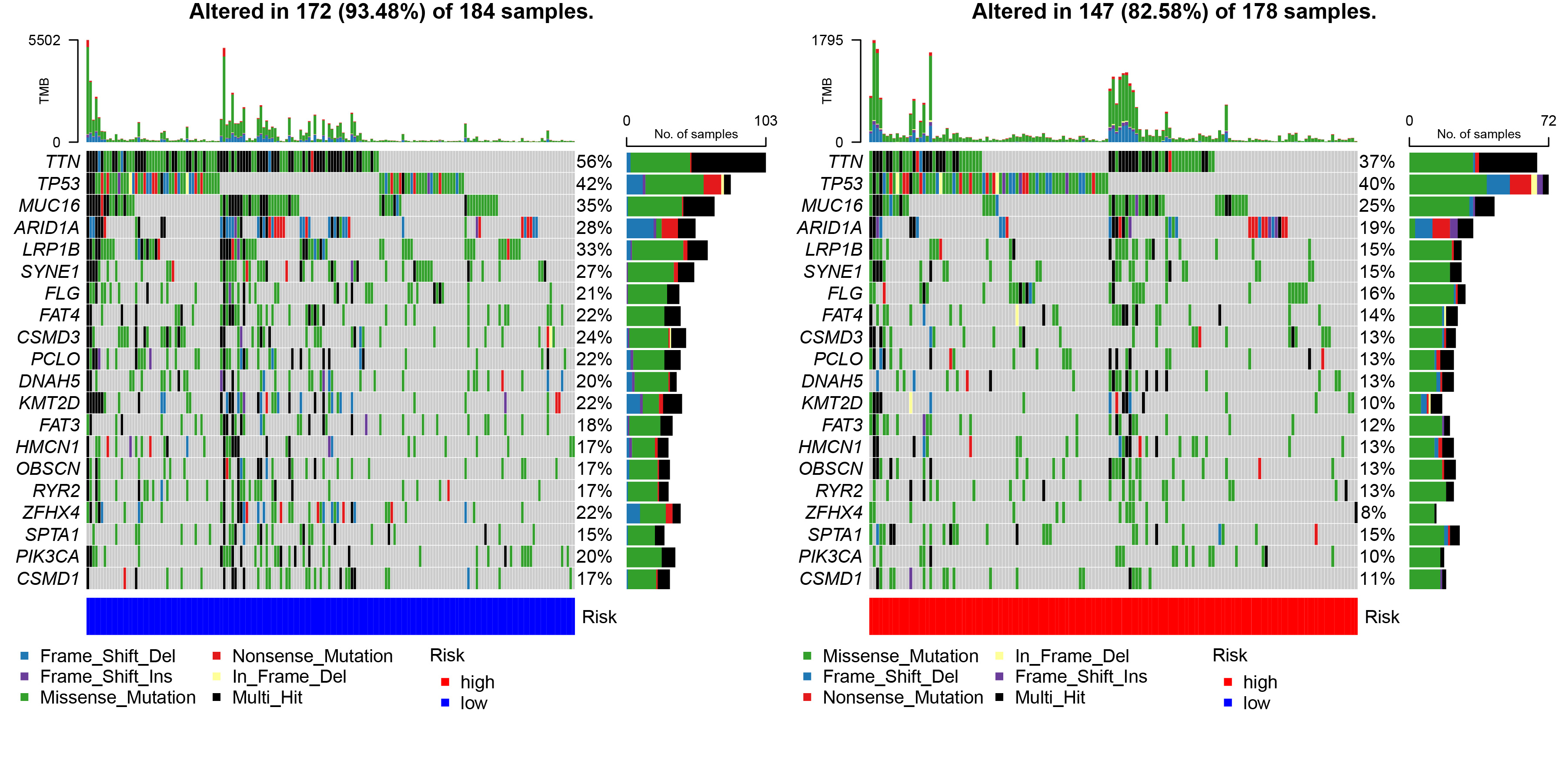

Supplement: Supplementary file 7 [file Image5.JPEG]
