# Supplementary material for: Identification of a novel reactive oxygen species (ROS)-related genes model combined with RT-qPCR experiments for prognosis and immunotherapy in gastric cancer
Source: Front Genet. 2023 Apr 14;14:1074900. doi: 10.3389/fgene.2023.1074900 (PMC10141461; doi:10.3389/fgene.2023.1074900)
Supplement: Supplementary file 3 [file DataSheet3.DOCX]

| Gene | F | R |
| --- | --- | --- |
| β-actin | TGACGTGGACATCCGCAAAG | CTGGAAGGTGGACAGCGAGG |
| GPX3 | ATCCGTGTCTCCAACCACAC | GCCTGGCAGTACACAGAACT |
| DUSP1 | ACCACCACCGTGTTCAACTT | GGGATGTGAAGAGCCTCACC |
| NOS3 | GAGGATGTGGCTGTCTGCAT | GGCTAGCTGGTAACTGTGCA |
| TCIRG1 | GCTGGGACTCTTCGGTTACC | GGCTGTGGGAGAAGAGGAAC |
