# Supplementary material for: Identification of a novel reactive oxygen species (ROS)-related genes model combined with RT-qPCR experiments for prognosis and immunotherapy in gastric cancer
Source: Front Genet. 2023 Apr 14;14:1074900. doi: 10.3389/fgene.2023.1074900 (PMC10141461; doi:10.3389/fgene.2023.1074900)
Supplement: Supplementary file 8 [file DataSheet2.DOCX]

| **Characteristics** | **TCGA-STAD** | **GSE84437** |
| --- | --- | --- |
| Number of samples | 375 | 433 |
| Median survival time (days) | 475 | 2,040 |
| Number of deaths, n (%) | 146 (41.71) | 209 (48.27) |
| Average age (years) | 65.25 | 60.06 |
| Gender, n (%) |  |  |
| Male | 226 (64.57) | 296 (68.36) |
| Female | 124 (35.43) | 137 (39.14) |
| FIGO stage, n (%) |  |  |
| I | 46 (13.14) | NA |
| II | 110 (31.43) | NA |
| III | 145 (41.43) | NA |
| IV | 35 (10.00) | NA |
| NA | 14 (4.00) | NA |
| Grade, n (%) |  |  |
| 1 | 9 (2.57) | NA |
| 2 | 125 (35.71) | NA |
| 3 | 207 (59.14) | NA |
| NA | 9 (2.57) | NA |
