# Supplementary material for: Identification of a novel reactive oxygen species (ROS)-related genes model combined with RT-qPCR experiments for prognosis and immunotherapy in gastric cancer
Source: Front Genet. 2023 Apr 14;14:1074900. doi: 10.3389/fgene.2023.1074900 (PMC10141461; doi:10.3389/fgene.2023.1074900)
Supplement: Supplementary file 9 [file DataSheet1.DOCX]

| GABRA1 |
| --- |
| GABRA2 |
| GABRA3 |
| GABRA4 |
| GABRA5 |
| GABRA6 |
| GPX1 |
| PRKCE |
| SOD1 |
| GCLC |
| GCLM |
| GGT1 |
| GPX2 |
| GPX3 |
| GSR |
| GSTA2 |
| GSTA3 |
| GSTA4 |
| GSTM1 |
| GSTM2 |
| GSTM3 |
| GSTM4 |
| GSTM5 |
| GSTP1 |
| GSTT1 |
| GSTZ1 |
| IDH1 |
| MGST2 |
| MGST3 |
| NCF1C |
| ATOX1 |
| CAT |
| CDO1 |
| DUSP1 |
| GOT1 |
| GPX4 |
| MPO |
| NUDT1 |
| PDLIM1 |
| PRDX6 |
| SDS |
| SELENOP |
| ATP6V0A1 |
| ATP6V0A2 |
| ATP6V0A4 |
| ATP6V0B |
| ATP6V0C |
| ATP6V0D1 |
| ATP6V0D2 |
| ATP6V0E1 |
| ATP6V0E2 |
| ATP6V1A |
| ATP6V1B1 |
| ATP6V1B2 |
| ATP6V1C1 |
| ATP6V1C2 |
| ATP6V1D |
| ATP6V1E1 |
| ATP6V1E2 |
| ATP6V1F |
| ATP6V1G1 |
| ATP6V1G2 |
| ATP6V1G3 |
| ATP6V1H |
| CYBA |
| CYBB |
| HVCN1 |
| LPO |
| NCF1 |
| NCF2 |
| NCF4 |
| NOS1 |
| NOS2 |
| NOS3 |
| RAC2 |
| SLC11A1 |
| TCIRG1 |
| CYP2E1 |
| MAFF |
| MAFG |
| MAFK |
| MAP2K1 |
| MAP2K2 |
| MAPK8 |
| NFE2L2 |
| PRKCQ |
| SP1 |
